# The Use of Artificial Gel Forming Bolalipids as Novel Formulations in Antimicrobial and Antifungal Therapy

**DOI:** 10.3390/pharmaceutics11070307

**Published:** 2019-07-01

**Authors:** Nathalie Goergen, Matthias Wojcik, Simon Drescher, Shashank Reddy Pinnapireddy, Jana Brüßler, Udo Bakowsky, Jarmila Jedelská

**Affiliations:** 1 Department of Pharmaceutics and Biopharmaceutics, University of Marburg, 35037 Marburg, Germany; 2 Institute of Pharmacy, Biophysical Pharmacy, Martin Luther University Halle-Wittenberg, 06120 Halle (Saale), Germany; 3 Institute of Pharmacy, University of Greifswald, 17489 Greifswald, Germany

**Keywords:** hydrogel, drug delivery system, self-assembly, bolaform amphiphilic lipids, bolalipids, aerogel, chorioallantoic membrane model, antimicrobial photodynamic therapy

## Abstract

The alarming growth of multi-drug resistant bacteria has led to a quest for alternative antibacterial therapeutics. One strategy to circumvent the already existing resistance is the use of photodynamic therapy. Antimicrobial photodynamic therapy (aPDT) involves the use of non-toxic photosensitizers in combination with light and in situ oxygen to generate toxic radical species within the microbial environment which circumvents the resistance building mechanism of the bacteria. Hydrogels are used ubiquitously in the biological and pharmaceutical fields, e.g., for wound dressing material or as drug delivery systems. Hydrogels formed by water-insoluble low-molecular weight gelators may potentially provide the much-needed benefits for these applications. Bolalipids are a superior example of such gelators. In the present work, two artificial bolalipids were used, namely PC-C32-PC and Me_2_PE-C32-Me_2_PE, which self-assemble in water into long and flexible nanofibers leading to a gelation of the surrounding solvent. The aim of the study was to create stable hydrogel formulations of both bolalipids and to investigate their applicability as a novel material for drug delivery systems. Furthermore, methylene blue—a well-known photosensitizer—was incorporated into the hydrogels in order to investigate the aPDT for the treatment of skin and mucosal infections using a custom designed LED device.

## 1. Introduction

Inappropriate use of antibiotics in humans and agriculture in recent decades has led to a rapid development of multi-drug resistant bacteria. According to WHO, antibiotic resistance is one of the biggest threats to global health, food security, and development, leading to longer hospital stays, higher medical costs and increased mortality [1]. Therefore, research into novel strategies to combat bacterial infections have become highly relevant. Photodynamic activity of chemical compounds towards bacterial microorganisms has been effective in the treatment of localized microbial infections [2]. The use of non-toxic photosensitizers in combination with light and in situ oxygen generates toxic radical species in the microbial environment [3,4]. Due to the unselective mechanism of action, antimicrobial photodynamic therapy (aPDT) has a broad spectrum of activity. In previous studies, it could be shown that antibiotic-resistant strains such as methicillin-resistant *Staphylococcus aureus* are as sensitive towards aPDT as non-resistant *Staphylococcus aureus* [4]. However, PDT is not only used in antibacterial research, but also in antifungal therapy [5,6]. In contrast to the PDT of tumors in which the photosensitizer is injected intratumorally or intravenously and is afterwards accumulated in the tumor tissue, the photosensitizer in aPDT is applied locally to the infected area, thereby making aPDT particularly suitable for the treatment of skin and soft tissue infections, such as burns or ulcus cruris [3,7].

For the local treatment, a drug delivery system (DDS) is necessary to deliver the drug in a controlled manner. Hydrogels have been used previously as DDS or wound dressing materials and possess most of the desirable characteristics [8,9,10]. They offer a moist wound environment, absorb blood and exudate and are suitable for cleansing of dry, sloughy or necrotic wounds by rehydrating dead tissues and enhancing the autolytic debridement [10]. Nevertheless, complications such as non-biodegradability, unfavorable mechanical properties or low biocompatibility are well-known limitations of the hydrogel technology in general [11].

Low biocompatibility is caused by the use of toxic cross-linkers or remaining unreacted monomers, oligomers and initiators of the hydrogel itself [12]. A broad variety of gelling agents have been described whereas low-molecular weight gelators have shown great potential as controlled DDS [13]. Within this expanding field of low-molecular weight gelators, lipids, which can self-assemble in hydrophilic media and thus form hydrogels, have garnered much interest [13]. A promising class among them are bipolar lipids, the so-called bolalipids. Bolalipids are defined as molecules that possess hydrophobic repeating units connecting hydrophilic head groups at the two ends of the hydrophobic core [14]. Both head groups can be identical (symmetric bolalipids) or they can differ in their size, charge, polarity and/or ability to (de) protonate (asymmetric bolalipids) [15,16,17,18]. Natural bolalipids originated from the membrane lipids of certain species of Archaea, especially from those of thermoacidophiles [19]. Archaea, apart from bacteria and eukaryotes, represent one of the three domains of life [20]. The organism Archaea thermoacidophiles is commonly found in exceptional ecological niches with high temperature of about 90 °C and a low pH of 2. These life circumstances result in the need of stable membranes, which make the bolalipids an interesting choice for new, innovative DDS. Previous work has shown that these naturally occurring lipids are well suitable to stabilize DDS [21,22,23,24,25].

Due to the fact, that the isolation of archaeal bolalipids from natural sources is expensive and often leads to a mixture of bolalipids with different alkyl chain pattern and head groups, artificial bolalipids offer an advantageous alternative [22,26,27]. A special class of artificial bolalipids, the single-chained ones, are able to form hydrogels by self-assembly [28]. This capacity is based on their ability to form an extended network of entangled helical nanofibers of 6–7 nm thickness depending on the concentration of bolalipid and the pH of the dispersion medium [28,29,30]. The self-assembly into nanofibers is mainly driven by hydrophobic (van-der-Waals) interactions of the long, single alkyl chain. In some cases, depending on the structure of the head group, these fibers are stabilized by hydrogen bonds. Finally, the fibers interact with the surrounding medium (water) also by means of hydrogen bonds, which leads to an efficient gelation of the solvent.

**Figure 1:**
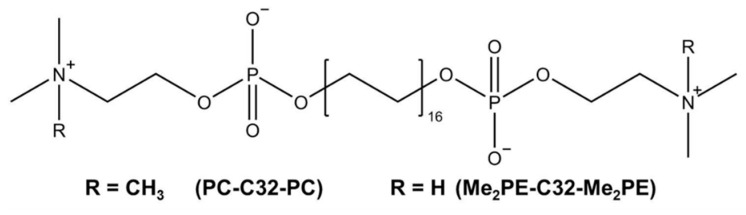
Chemical structure of bolalipids PC-C32-PC and Me_2_PE-C32-Me_2_PE used in this study.

In the present work, we focused on two artificial bolalipids, namely PC-C32-PC and Me_2_PE-C32-Me_2_PE, which are composed of a long C32 alkyl chain and either two phosphocholine (PC) or dimethylphosphoethanolamine (Me_2_PE) head groups. The chemical structures are depicted in Figure 1. Previous characterizations of the investigated bolalipids demonstrated gel formation with concentrations lower than 1 mg/mL [31,32] which makes them highly interesting as material for the use in novel DDS.

The aim of the study was to create stable formulations of both bolalipids and to investigate their applicability as a novel material for DDS. Furthermore, methylene blue (MB), a well-known photosensitizer [33,34,35,36] was incorporated into the formulations in order to consider the application of these formulations in aPDT for the treatment of skin and mucous infections.

For the characterization of all formulations, rotation viscometry as well as scanning electron microscopy was used. Drug release was performed using Franz diffusion cells. In addition to physical characterization of the bolalipid formulations, the biological aspects of DDS requirements were investigated. The ability of PC-C32-PC and Me_2_PE-C32-Me_2_PE to inhibit microbial growth of bacteria and fungi itself was examined by means of agar diffusion method [37,38]. To investigate the compliance on mucous tissue, the bolalipid formulations were topically applied on the chorioallantoic membrane (CAM) model and structural changes of the blood vessels were investigated [39].

## 2. Materials and Methods

### 2.1. Materials

The bolalipids dotriacontane-1,1′-diylbis [2-(trimethylammino)ethyl phosphate] (PC-C32-PC) and dotriacontane-1,1′-diylbis [2-(dimethylammino)ethyl phosphate] (Me_2_PE-C32-Me_2_PE) were synthesized at the MLU Halle-Wittenberg (Halle, Germany) according to procedures described previously [40,41]. Sodium dodecyl sulfate and methylene blue were purchased from Carl Roth GmbH and Co. KG (Karlsruhe, Germany). Hydroxyethyl cellulose 300 (HEC 300; M_w_ ~ 807 g/mol) was obtained from Caesar and Loretz GmbH (Hilden, Germany). Physiological saline solution was purchased from B Braun (Melsungen, Germany). *Saccharomyces cerevisiae* were obtained from a retail outlet. Clinically isolated *Staphylococcus aureus* (ATCC 25923) was determined using MALDI Biotyper (Bruker Corporation, Billerica, MA, USA). Fertilized chicken eggs were obtained from Brormann GmbH (Rheda-Wiedenbrück, Germany). For all experiments ultrapure water from PURELAB^®®^ flex 4 (ELGA LabWater, High Wycombe, UK) was used.

### 2.2. Preparation of Hydrogels and Aerogels

A defined amount of 5 mg/mL PC-C32-PC or Me_2_PE-C32-Me_2_PE was dissolved in ultrapure water in a 100 °C water bath. After cooling to room temperature, gelation occurred. For the release studies, MB was dissolved in the water prior to gelation. To compare the bolalipids with a commonly used hydrogel, 5 mg/mL HEC 300 dissolved in ultrapure water was used. To obtain aerogels, 75 µL of the hydrogels were pipetted into 96-well plates, frozen at −20 °C and transferred to a freeze dryer (ALPHA 1–4 LSC, Martin Christ Gefriertrocknungsanlangen GmbH, Osterode, Germany). The aerogels were stored under dry conditions after the preparation. For the rheological measurements, the aerogels were rehydrated in ultrapure water and stored at 37 °C.

### 2.3. Characterization

#### 2.3.1. Rheological Characterization of Hydrogels and Rehydrated Aerogels

Rheometry was performed using a Haake^TM^ Rotovisco 1 (Thermo Scientific, Karlsruhe, Germany) in the C/R ramp mode with cone/plate geometry of 60 mm in diameter and a slit of 0.54 mm. For each measurement, a sample volume of 2 mL was necessary to fill the slit. Before the measurement started, a recovery time of 10 min was applied after the slit size was reached. The recovery time was essential to make sure that the pre-stressed sample relaxed. The temperature was kept constant at 20 °C during the experiment, which was performed in triplicate for each substance.

#### 2.3.2. Scanning Electron Microscopy (SEM) of Aerogels

To investigate the structure of bolalipid aerogels, SEM pictures were generated using Hitachi S-510 scanning electron microscope (Hitachi-High Technologies Europe GmbH, Krefeld, Germany) under a high vacuum of 4 × 10^−6^ mbar at 5 kV accelerating voltage and 30 µA emission current [42]. The aerogels as well as freeze-dried HEC 300 hydrogels were sputter coated with gold at 1.3 × 10^−1^ mbar vacuum under argon atmosphere for 1 min at 30 mA (Edwards S150, Edwards High Vacuum, Crawley, UK) [43]. This procedure ensures a sufficient conductivity of organic material, increases the signal of emitted electrons and leads to higher resolution of obtained pictures.

### 2.4. Release Studies

The release behaviors of bolalipid hydro- and aerogels were investigated using a vertical diffusion cell with a release area of approx. 180 mm^2^ and a volume of 12 mL equipped with a 0.22 µm membrane filter. Acceptor chamber was filled with physiological buffer and the temperature was maintained at 37.8 °C [44]. As reference, HEC 300 containing the same concentration of gelling agent and drug was used. After placing the hydro- or aerogels into the donor chamber, samples were collected from the acceptor chamber at defined time points. The concentration of MB was calculated using microplate spectrometer (Multiskan™ GO, Thermo Scientific, Waltham, MA, USA) at a wavelength of 664 nm. The experiment was conducted with six diffusion cells for each formulation.

### 2.5. Microbiology

#### 2.5.1. Antifungal Activity of PC-C32-PC and Me_2_PE-C32-Me_2_PE Hydro- or Aerogels

To investigate the antifungal effect of bolalipid hydrogels and aerogels, the agar diffusion test was used as described previously [37,38]. In brief, Mueller Hinton agar plates (BD GmbH, Heidelberg, Germany) were inoculated with a suspension of *Saccharomyces cerevisiae*. The samples containing PC-C32-PC or Me_2_PE-C32-Me_2_PE bolalipids were placed on the inoculated agar plates. After incubation overnight (Heraeus GmbH and Co. KG., Hanau, Germany) at 30 °C and 60% relative humidity, the antifungal activity was evaluated.

#### 2.5.2. Antifungal Activity of Loaded PC-C32-PC and Me_2_PE-C32-Me_2_PE Aerogels by Means of PDT

Antifungal activity of bolalipid aerogels loaded with MB were investigated using PDT. The agar plates were inoculated in the same manner as described above (2.5.1). Then the loaded aerogels were placed on the inoculated agar plates. After an incubation time of 3 h, the complete agar plate was irradiated (λ_ex_ = 643 nm) using a custom designed LED device (Lumundus GmbH, Eisenach, Germany) for 20 min resulting in a radiation fluence of 26.88 J/cm^2^. Further technical characteristics of the device were described before [45]. Non-irradiated, loaded bolalipid aerogels as well as empty aerogels and MB solution served as controls. Subsequently, the plates were returned into the incubator and incubated overnight at 30 °C and 60% relative humidity. After 18 h, the antifungal effect was evaluated. The experiments were performed in triplicate.

#### 2.5.3. Antibacterial Activity of Loaded PC-C32-PC and Me_2_PE-C32-Me_2_PE Aerogels by Means of aPDT

The aPDT studies were carried out on clinically isolated *Staphylococcus aureus* using the agar diffusion test. Briefly, bacterial suspension was prepared performing the direct colony suspension method. Afterwards a sterile cotton swap was dipped into the suspension and the agar plates (BD Columbia agar, BD GmbH, Heidelberg, Germany) were inoculated. In a similar manner to the antifungal PDT experiments, the samples were placed on the surface of inoculated agar plates and after an incubation time of 3 h the whole area was irradiated (λ_ex_ = 643 nm) using the LED device (radiation fluence 26.88 J/cm^2^). Non-irradiated, loaded bolalipid aerogels as well as empty aerogels were used as controls. Finally, the treated plates were returned to the incubator and incubated overnight at 37 °C and 5% CO_2_. After 20 h, the antimicrobial effect was evaluated. The experiments were performed in triplicate.

### 2.6. Biocompatibility

To evaluate the biocompatibility of the bolalipid hydro- and aerogels, the CAM model was used. Upon delivery, fertilized chicken eggs were swabbed with 70% (*v*/*v*) ethanol and incubated at 37.8 °C and 60–70% relative humidity in a hatching incubator (Ehret KMB 6, Ehret GmbH, Emmendingen, Germany). Until egg development day (EDD) 4, the eggs were rotated all 4 h automatically. On EDD 4, a window (⌀ 3 cm) at the broad pole was opened using a pneumatic egg opener (Schuett-Biotec GmbH, Göttingen, Germany). The eggs were then returned to the incubator [46].

#### 2.6.1. Henn´s Egg Test on the Chorioallantoic Membrane (HET-CAM) of Hydrogels

On EDD 10, 300 µL of PC-C32-PC, Me_2_PE-C32-Me_2_PE or HEC 300 hydrogels were applied onto the CAM surface and observed under a stereomicroscope (Stemi 2000-C, Carl Zeiss Microscopy GmbH, Göttingen, Germany) at 13-fold magnification. Supplied shear stress during the pipetting procedure led to liquefaction of bolalipid hydrogels. As a positive control, 1% (*w*/*w*) sodium dodecyl sulfate (SDS) and as a negative control, physiological saline solution were used. Pictures were taken each minute beginning with the contact of the sample to the CAM surface up to 5 min using a digital camera connected to the stereomicroscope (Moticam 2000, Motic Deutschland GmbH, Wetzlar, Germany). Each image was captured using the Motic Image Plus 2.0 software, followed by calculation of the irritation score as described elsewhere previously [47]. The experiment was conducted with six eggs for each substance.

#### 2.6.2. Modified HET-CAM Assay with Bolalipid Aerogels

To evaluate the long-term biocompatibility of bolalipid aerogels, PC-C32-PC or Me_2_PE-C32-Me_2_PE aerogels were carefully placed on the CAM surface at EDD 9. A daily analysis of the samples, vessel formation and health of the CAM surface was carried out at 13-fold magnification using a stereomicroscope [48,49]. Pictures were taken as described above. The experiment was performed with five eggs for each bolalipid aerogel.

## 3. Results and Discussion

The aim of the study was to develop a stable and applicable DDS for aPDT based on the bolalipids PC-C32-PC and Me_2_PE-C32-Me_2_PE. Previous characterizations were performed using 1 mg/mL bolalipid resulting in a low stability of the hydrogels of this concentration [31,32]. Expecting an increase in the macroscopic mechanical stability, for a perspective pharmaceutical application, the concentration of bolalipids was elevated to 5 mg/mL.

### 3.1. Characterization of Hydrogels and Aerogels

**Figure 2:**
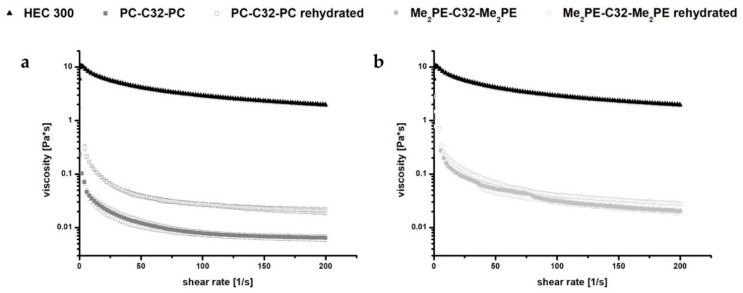
Rheological measurements of 5 mg/mL (**a**) PC-C32-PC and (**b**) Me_2_PE-C32-Me_2_PE bolalipid hydrogels and rehydrated aerogels, respectively, compared to HEC 300 hydrogels.

After dispersion of 5 mg/mL bolalipid (PC-C32-PC or Me_2_PE-C32-Me_2_PE) in ultrapure water, a hydrogel formation has been reported [28,30]. The hydrogels were thus characterized by rotational viscometry (Figure 2). The obtained rheological data were compared to those of 5 mg/mL HEC 300 hydrogels as a reference. The measurements revealed that the viscosity of bolalipid hydrogels decreased at minimal shear forces. The low stability of these hydrogels in contrast to HEC 300 could be explained with weak interactions between the hydrophobic chains of the bolalipids (van-der-Waals only) and fewer amount of hydrogen bonds between the nanofibers [50]. Minimal shear forces thus led to a disintegration of the weakly bond nanofibers. This disintegration goes along with the rapid decrease of the viscosity and loss of the gel structure.

**Figure 3:**
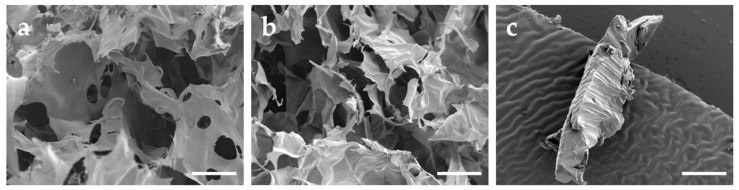
SEM images of (**a**) PC-C32-PC aerogel, (**b**) Me_2_PE-C32-Me_2_PE aerogel, and (**c**) freeze-dried HEC 300 hydrogel. Scale bar represents 100 µm.

To optimize the stability of the obtained bolalipid hydrogels, the incorporated mobile phase was sublimated using a conventional freeze dryer. All samples were lyophilized under the same conditions. During this process, the hydrogels were transformed into bolalipid aerogels. These more stable and applicable DDS were characterized by SEM (Figure 3). Whereas obtained bolalipid aerogels retained a porous appearance (Figure 3a,b), the HEC 300 formulation displayed a compact appearance (Figure 3c). Due to the porous structures of the bolalipid aerogels, they were able to reform hydrogels in situ after addition of liquid.

The rheological behavior of rehydrated bolalipid aerogels was compared with the rheological properties of the bolalipid hydrogels. Obtained data indicate a slightly different behavior of rehydrated PC-C32-PC aerogel compared to its hydrogel. The rehydrated aerogel displayed a low increase of viscosity. In the case of Me_2_PE-C32-Me_2_PE, the rehydrated aerogel showed nearly the same rheological characteristics as compared to its hydrogel (see Figure 2).

### 3.2. Release Studies

A suitable and controlled drug release is an essential part of a DDS. The drug release rate depends on several factors, e.g., application site, porosity and degradation of DDS as well as exposed surface area. The ability of hydrogel systems to release incorporated substances has been extensively reported and mathematically described in the last years [51,52,53]. Recent research suggests three types of release: diffusion-controlled, chemically-controlled or swelling-controlled. However, all systems are mainly based on the diffusion of the therapeutic agent out of the bulk system. Furthermore, the phenomenon of diffusion is closely connected to the structure of the material through which the diffusion takes place [12].

**Figure 4:**
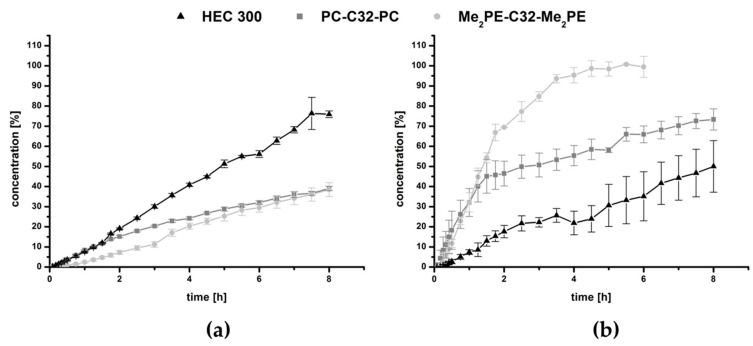
Drug release of (**a**) hydrogels and (**b**) aerogels using PC-C32-PC, Me_2_PE-C32-Me_2_PE and HEC 300, respectively.

In the present study, MB was used as a model drug. MB has several indications in medicine; it has been used to treat methemoglobinemia, malaria and urinary tract infections. During the last two decades, MB has attracted interest as a photosensitizer especially in cancer research [35,36,54]. Recently, MB has become increasingly important in antimicrobial studies [54,55]. An amount of 3.8 mg/mL of MB was distributed in the hydrogels. The results of the release studies are plotted in Figure 4.

Over the time period of 8 h, both bolalipid hydrogels released less than 40% of MB, while the reference with the same amount of gelling agent HEC 300 released approximately 75% (Figure 4a). This property could be explained by the structure of the bolalipid hydrogel: The hydrogel is formed due to the entanglement of nanofibers built up by the bolalipids [28,29]. Within these nanofibers, the bolalipid molecules are arranged side-by-side but twisted relative to each other due to the bulky PC or Me_2_PE headgroup compared to the small cross-sectional area of one single alkyl chain. This twist leads to a helical superstructure of the nanofibers with hydrophobic grooves that are exposed to the surrounding water [56]. Hydrophobic and amphiphilic substances can now interact with these grooves, which was previously shown for cholesterol [57], phospholipids such as dipalmitoylphosphatidylcholine [58], and the fluorescence probe bis-ANS [59]. It is therefore conceivable that also MB, carrying a phenothiazine core, interacts with these hydrophobic grooves of the bolalipid nanofibers. In contrast, HEC 300 is a nonionic and hydrophilic gelling agent, which is not able to form hydrophobic interactions. Hence, the release of MB is lower and slower when bolalipid hydrogels are compared to the HEC 300 hydrogel. Comparing both bolalipids, the release of Me_2_PE-C32-Me_2_PE was slightly lower than those of PC-C32-PC. This is due to the ability of Me_2_PE-C32-Me_2_PE to form additional hydrogen bond to MB, using the proton at the quarternary amine, which is absent in PC-C32-PC bolalipid.

The release profile changed dramatically after the lyophilization process. In Figure 4b, the results showed a burst release of all bolalipid aerogels in contrast to the freeze-dried HEC 300, which displayed approximately a linear release rate. Within the first 1.5 h, the release rate of both bolalipid aerogels presented nearly the same behavior because it was mainly controlled by the fast diffusion of the MB out of the bolalipid aerogel framework. The water influx into the aerogels led to the formation of hydrogels. For PC-C32-PC, this re-forming could be observed within a few hours; whereas in the case of Me_2_PE-C32-Me_2_PE aerogels, it took several days to transform to a hydrogel again. The release from the PC-C32-PC aerogel seemed to be swelling-controlled after 1.5 h. For the Me_2_PE-C32-Me_2_PE aerogel, the release remained very fast after 1.5 h, indicating a rapid diffusion of the drug out of the aerogel. Comparing both bolalipid aerogels with lyophilized HEC 300, a higher amount of released MB was measured. Since the release rate from aerogels is determined by several factors, such as the interaction of MB with the gelling agent in the dry state and the velocity of the water influx into the aerogel (and the re-formation of the hydrogel), an interpretation of the data is difficult. Nevertheless, to explain this release behavior, the following scenario is conceivable. As mentioned before, hydrophobic interactions between MB and the hydrophobic grooves of the bolalipid nanofibers led to the slow release of MB from the bolalipid hydrogels. These hydrophobic interactions consist of an enthalpic contribution (van-der-Waals interactions) and an entropic contribution, i.e., the release of bound water molecules. In the case of an aerogel, the entropic contributions to the hydrophobic interactions are missing, since no water is present in the aerogel. Hence, the remaining van-der-Waals interactions between MB and the bolalipid aerogel are much weaker compared to the hydrogen bond interactions between MB and the HEC 300 aerogel, leading to a faster release of MB from the bolalipid aerogels. The higher release rate from Me_2_PE-C32-Me_2_PE aerogel compared to the PC-C32-PC counterpart could be explained by the slower influx of water. Furthermore, the structure of the obtained bolalipid aerogels was completely different when compared to the lyophilized HEC 300 hydrogel. Both bolalipid aerogels showed a high number of pores (Figure 3a,b) whereas no pores could be found in the HEC 300 sample (Figure 3c), which led to higher exposed area of the aerogel framework to the surrounding medium. This could explain the different release behaviors of the freeze-dried systems. Additionally, the different sizes of the error bars plotted in the graphs (Figure 4) could be elucidated with the fact that the drug release rate is strongly dependent on the exposed area of the DDS to the acceptor medium: Hydrogels are viscous and hence spread quickly over the entire area of the donor chamber, whereas the aerogels change their contact area quite slowly and differently for each sample leading to larger error bars.

### 3.3. Microbiology

Antimicrobial resistance is not a new phenomenon, but the impact of antibiotics to kill bacteria efficiently has dramatically decreased during the last years [60,61]. The ability of bacteria to develop appropriate resistance against a high amount of antibiotics in short time led to challenges in antimicrobial therapy. An antimicrobial activity of the DDS itself, would offer huge benefits. We investigated the ability of both bolalipids to inhibit the growth of fungi as well as bacteria, using different methods.

#### 3.3.1. Antifungal Activity of PC-C32-PC and Me_2_PE-C32-Me_2_PE Hydro- or Aerogels

**Figure 5:**
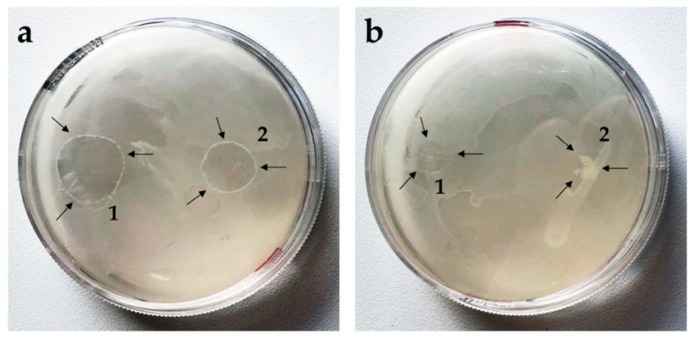
Agar diffusion test on *Saccharomyces cerevisiae*. Arrows indicate the bolalipid (**a**) hydrogels and (**b**) aerogels of (1) PC-C32-PC and (2) Me_2_PE-C32-Me_2_PE.

Previous studies have shown that PC-C22-PC (Irlbacholine), a bolalipid with shorter alkyl chain, which can be found in *Irlbachia alata* and *Anthocleista djalonensis*, is effective against the fungus *Trichophyton rubrum* and other fungal infections [62,63]. To investigate the antifungal activity of both bolalipid hydro- and aerogels, the agar diffusion test [37,38] was performed. *Saccharomyces cerevisiae* was used as model yeast. To compare the ability of the formulations to inhibit the growth of the model yeast, aerogels as well as hydrogels (75 µL) were applied on inoculated agar plates. After 24 h, fungal growth on the agar plate was observed. While PC-C32-PC hydrogel spread out to a broad shape (Figure 5a1), Me_2_PE-C32-Me_2_PE hydrogel retained more or less its original form (Figure 5a2). Comparing the aerogel-formulations of both lipids, in the case of the PC-C32-PC, a fast rehydration to hydrogel took place (Figure 5b1), whereas the Me_2_PE-C32-Me_2_PE aerogel was not able to reform hydrogel during the incubation time. However, in all cases, the inhibition zone was not evident. This indicates that the examined bolalipids itself did not possess any antifungal activity.

#### 3.3.2. Antifungal Activity of Loaded PC-C32-PC and Me_2_PE-C32-Me_2_PE Aerogels by Means of PDT

Superficial skin mycosis is one of the most frequent diseases in human beings which is mainly caused by dermatophytes which exhibit increasing rates of resistant strains [5,6]. The appearance of drug resistant strains is more and more frequent in immunocompromised individuals such as high-risk groups, e.g., HIV+ and cancer patients undergoing chemotherapy [5]. In dermatology, PDT has proven to be a useful treatment for a variety of selected inflammatory diseases [3,5] as well as fungal infections [6]. Previous studies demonstrated that *Candida albicans* and dermatophytes were effectively killed by MB solution in combination with light. *Saccharomyces cerevisiae* was chosen as model yeast to investigate the antifungal activity of both bolalipid aerogels. The results are summarized in Table 1. Non-irradiated methylene blue released from the aerogels did not affect the growth of the yeast significantly. Irradiation resulted in an occurrence of a characteristic area with absence of yeast growth. Yeasts incubated with unloaded aerogels showed natural growth regardless of irradiation (Supplementary Figure S1).

#### 3.3.3. aPDT with PC-C32-PC and Me_2_PE-C32-Me_2_PE Aerogels Containing Methylene Blue

As reported previously, *Staphylococcus aureus* is almost the universal cause of furuncles, carbuncles and skin abscesses and are worldwide the most commonly identified agent responsible for skin and soft tissue infection [64]. Earlier studies have shown that MB in combination with light reduced bacterial growth. In some clinical cases, an effective PDT using MB as a photosensitizer for skin ulcers could be demonstrated. This kind of therapy led to clinical and microbial cure with no significant adverse effects [54]. According to the data obtained from release studies and the antifungal activity tests, we decided to use only bolalipid aerogels loaded with MB for the aPDT experiment. Based on the data of Tardivo et al. the fluence was set to 26.88 J/cm^2^ [35].

**Figure 6:**
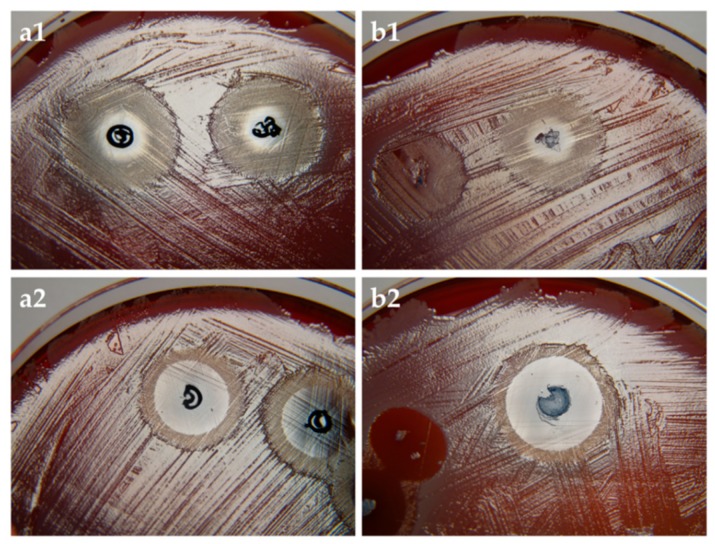
(**a**) PC-C32-PC and (**b**) Me_2_PE-C32-Me_2_PE aerogel containing MB on *Staphylococcus aureus*. (1) non-irradiated and (2) irradiated samples. The size of the inhibition zones of irradiated samples ranged between 13 mm and 17 mm.

From the results (Figure 6) it is clearly evident that MB affected the growth of *Staphylococcus aureus*. In the case of non-irradiated agar plates (Figure 6a1,b1), the zone with absence of bacterial growth occurred in the immediate surrounding of the aerogel. In the case of irradiated plates, more extensive effect was observed (Figure 6a2,b2). As expected, bacteria incubated with unloaded aerogels exhibited normal growth regardless of irradiation, thus demonstrating that light source alone had no toxic effect (Supplementary Figure S2). Comparing the size of the inhibition zone of both irradiated bolalipids, Me_2_PE-C32-Me_2_PE demonstrated a slightly larger zone of inhibition to that of PC-C32-PC, which could be explained by the release profile of the bolalipid aerogels (Figure 4b). As mentioned before, Me_2_PE-C32-Me_2_PE showed a higher burst release comparing to PC-C32-PC. During the incubation time of 3 h, more MB was released out of the Me_2_PE-C32-Me_2_PE aerogel resulting in a higher aPDT effect.

### 3.4. Biocompatibility

As mentioned before, aPDT is well suitable for the treatment of skin, soft tissue and mucosal infections [3,7]. Therefore, DDS used for aPDT should exhibit a favorable biocompatibility. Preclinical assays applying mammalian models are still time-consuming and controversial [65]. Conventional in vivo tests are time- and labor-intensive as well as expensive. The CAM model allows an uncomplicated, economical and fast procedure with results comparable to mammalian models [66].

#### 3.4.1. HET-CAM of Bolalipid Hydrogels

**Figure 7:**
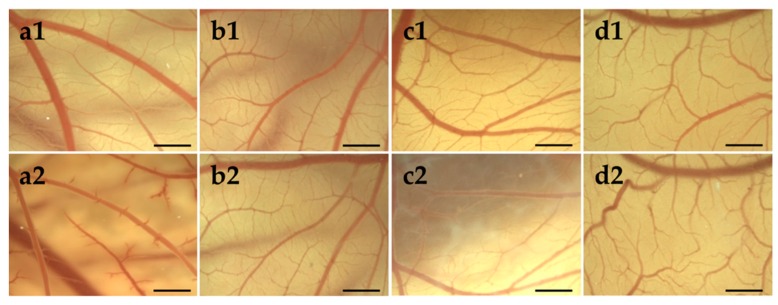
Stereomicroscopic images of chorioallantoic membrane (CAM) on egg development day (EDD) 10 (1) before and (2) 5 min after the treatment with (**a**) 1% (*w*/*w*) sodium dodecyl sulfate (SDS) solution, (**b**) physiological saline solution, (**c**) PC-C32-PC hydrogel and (**d**) Me_2_PE-C32-Me_2_PE hydrogel. Scale bar represents 1 mm.

Mucosa compatibility study of hydrogels was performed using HET-CAM test [67], which replaced the Draize rabbit eye test [68]. During the experiment, the occurrence of hemorrhage, coagulation and lysis was monitored, and the irritation scores (IS) were determined. The results of HET-CAM assay, shown in Figure 7 indicated no irritation potential of PC-C32-PC and Me_2_PE-C32-Me_2_PE, respectively. During the treatment with 1% (*w*/*w*) SDS solution (positive control; Figure 7a), CAM displayed multiple injuries resulting in an IS of 18.7 in average corresponding to “strong irritation assessment”. In contrast, an IS of 0 for each egg treated with both bolalipid hydrogels (Figure 7c,d) as well as physiological saline solution (negative control; Figure 7b), indicated “practically no” irritation assessment [69].

The PC-C32-PC bola lipid showed a remarkable characteristic on CAM surface: With progressing time, an increased rigidity of the applied dispersion was observed, which resulted in white streaks and opalescence as shown in Figure 7c2. This characteristic did not influence the vessels on the CAM surface. During the observation time of 5 min, this behavior did not occur in the case of the Me_2_PE-C32-Me_2_PE bolalipid.

#### 3.4.2. HET-CAM of Bolalipid Aerogels

Biocompatibility of bolalipid aerogels was investigated in long-term CAM assay. Beginning with EDD 9, the occurrence of hemorrhage, lysis, coagulation or angiogenesis was monitored daily until EDD 14. During the rehydration process of bolalipid aerogels, and the thereto related osmotic suction, micro vessels in the capillary plexus could be damaged.

**Figure 8:**
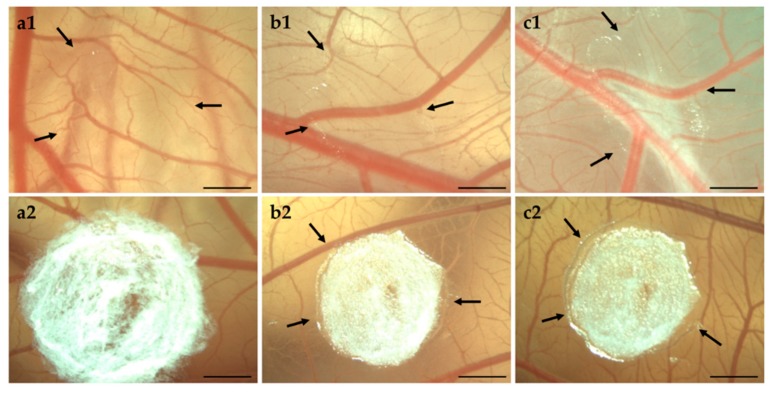
Stereomicroscopic images of CAM on (**a**) EDD 9, (**b**) EDD 12, and (**c**) EDD 14. The arrows indicate hydrogel formation of (1) PC-C32-PC aerogel and (2) Me_2_PE-C32-Me_2_PE aerogel. Scale bar represents 1 mm.

As shown in Figure 8, both bolalipid aerogels showed no irritation. It is clearly visible that PC-C32-PC aerogel rehydrated to hydrogel immediately after the placement on the CAM surface, while Me_2_PE-C32-Me_2_PE aerogels needed several days. The gelation process is indicated by arrows in Figure 8: It began from the border of bolalipid aerogels and progressed to the center of it. The different gelation processes of both bolalipids could be explained again with the chemical structure of PC-C32-PC and Me_2_PE-C32-Me_2_PE. As assumed before, due to hydrogen bond mediated stabilization of the head groups in the Me_2_PE-C32-Me_2_PE bolalipid aerogel, less hydrogen bonds can be assembled to the surrounding fluid.

## 4. Conclusions

The aim of the study was to create stable formulation of both bolalipids and to investigate their suitability as a novel material for a drug delivery system (DDS). It was detectable that the stability of the self-assembled hydrogels decreased with minimal shear forces. With the help of sublimation in a conventional freeze-drier, it was possible to create stable aerogels, which could be transformed into hydrogels by addition of liquid. The release studies demonstrated that all hydrogels showed sustained release, but bolalipid hydrogels were superior to HEC 300 hydrogel in terms of the release time. Nevertheless, the bolalipid aerogels showed a high burst release, which make them unsuitable as DDS in their native form. Combining the benefits of both systems seems to be more effective. Aerogels possess a long-term stability and easy-to-handle system for in situ hydrogel formation.

However, using these bolalipid aerogels that have methylene blue—a photosensitizer—incorporated, antimicrobial photodynamic therapy (aPDT) of *Staphylococcus aureus* as well as of *Saccharomyces cerevisiae* could be successfully demonstrated. Both formulations were able to inhibit the growth of bacteria and yeast. However, the treatment with light using the custom designed LED device led to an additional antimicrobial effect of bolalipid aerogels.

In the biocompatibility studies on the chorioallantoic membrane (CAM) surface, both bolalipid formulations showed an excellent biocompatibility and thus making them a potential material for DDS. Furthermore, in the biocompatibility studies on CAM surface of the hydrogels, it was clearly evident that both bolalipid hydrogels, especially PC-C32-PC, show a high solidification capacity under body temperature conditions.

These results make the PC-C32-PC and Me_2_PE-C32-Me_2_PE bolalipids an interesting novel material for DDS with a potential for the application in aPDT for the treatment of skin and mucosal infections.

## Figures and Tables

**Table 1:** Results of size measurements of the inhibition zones in mm (mean value).

Bolalipid	Sample	Non-Irradiated [mm]	Irradiated [mm]
PC-C32-PC	aerogel	4.5	4.7
	aerogel containing MB	5.3	13.7
Me_2_PE-C32-Me_2_PE	aerogel	4.0	4.7
	aerogel containing MB	6.7	15.3

## Supplementary Materials

The following Figure is available online at https://www.mdpi.com/1999-4923/11/7/307/s1, Figure S1: Negative control of bolalipid aerogels without MB in PDT on *Saccharomyces cerevisiae*, Figure S2: Overview of aPDT on *Staphylococcus aureus*.
