# Supplementary material for: The Use of Artificial Gel Forming Bolalipids as Novel Formulations in Antimicrobial and Antifungal Therapy
Source: Pharmaceutics. 2019 Jul 1;11(7):307. doi: 10.3390/pharmaceutics11070307 (PMC6680875; doi:10.3390/pharmaceutics11070307)
Supplement: Supplementary file 1 [file pharmaceutics-11-00307-s001.pdf]

pharmaceutics

Supplementary Materials: The Use of Artificial Gel Forming Bolalipids as Novel Formulations in Antimicrobial and Antifungal Therapy

Nathalie Goergen, Matthias Wojcik, Simon Drescher, Shashank Reddy Pinnapireddy, Jana Brüßler, Udo Bakowsky and Jarmila Jedelská

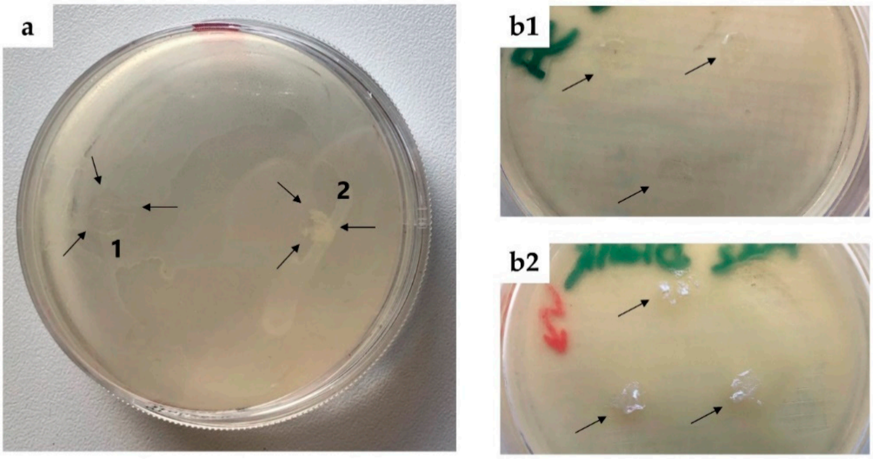

Figure S1a. Negative control of bolalipid aerogels without MB. PDT towards *Saccharomyces cerevisiae*. The arrows indicate (**a**) non-irradiated and (**b**) irradiated aerogels with (**1**) PC-C32-PC and (**2**) Me_2PE-C32-Me_2PE. In all case, no inhibition of yeast growth was detectable.

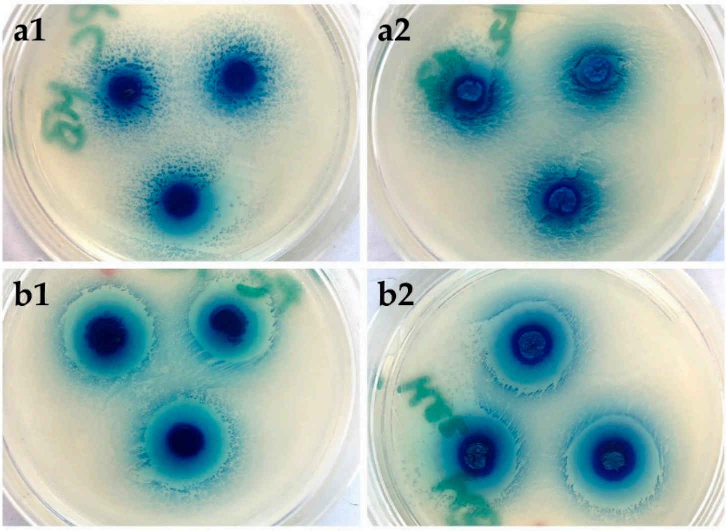

Figure S1b. PDT towards *Saccharomyces cerevisiae* with (**1**) PC-C32-PC and (**2**) Me_2PE-C32-Me_2PE bolalipid aerogels containing MB. Irradiated aerogels (**b1**,**b2**) exhibited larger inhibition zone compared to non-irradiated samples (**a1**,**a2**).

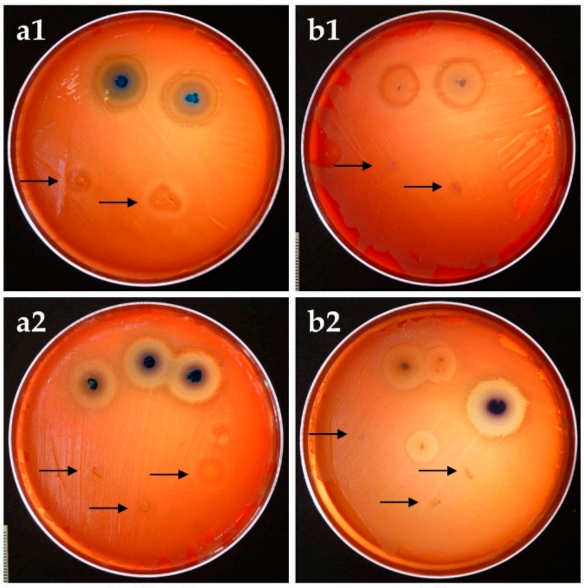

Figure S2. Overview of aPDT towards *Staphylococcus aureus*. (**a**) PC-C32-PC and (**b**) Me_2PE-C32-Me_2PE aerogels with (**1**) non-irradiated and (**2**) irradiated samples. Arrows indicate negative controls (aerogels without MB).
